# Caesarean sections in the in the context of the Chiranjeevi Yojana public private partnership program to promote institutional birth in Gujarat, India; does the embedded disincentive for caesarean section work?

**DOI:** 10.1186/s12939-019-0922-5

**Published:** 2019-01-24

**Authors:** Mariano Salazar, Kranti Vora, Kristi Sidney Annerstedt, Ayesha De Costa

**Affiliations:** 1 Department of Public Health Sciences, Karolinska Institutet, Tomtebodavägen 18a, Widerströmska Huset, 171 77 Stockholm, Sweden; 2 Department of Reproductive and Child Health, Indian Institute of Public Health, Gandhinagar, Ahmedabad, Gujarat India

**Keywords:** Cesarean section, India, Gujarat, Population-based, Cohort, Public private partnership, Financial incentive

## Abstract

**Background:**

The government of Gujarat, India runs a large public private partnership program to widen access to emergency obstetric care (EmOC). The program include a disincentive for Cesareans section (CS) which are capped at seven per 100 women. In this paper, we study if the disincentive works by comparing CS rates among similar groups of women who deliver within and outside the program.

**Methods:**

Community-based panel study in three districts of Gujarat, India. Sample size: 2123 women. Data was analyzed using multivariable logistic regression.

**Results:**

Overall seven point seven % (164/2123) of the all women in the study had a CS. After adjusting for confounding factors women within the program had 62% (AOR 0.38, 95% CI 0.22–0.44) lower odds of having a CS than to non-beneficiaries. In a separate model of predictors of CS among women giving birth only in program accredited hospitals, we found that CY program beneficiaries had lower odds of having a CS birth than non-beneficiary women (paying clients) (AOR 0.40, 95% CI 0.24–0.67).

**Conclusions:**

The Gujarat government is trying to ensure access to EmOC (including CS) for its vulnerable population through CY. The embedded disincentive to prevent unnecessary cesareans by private obstetricians is a novel one, and appears to work, though one could argue it works ‘over-efficiently’ by depriving some women who need CS from receiving one under the program. The state needs to revisit and review what is happening in the program periodically, and have oversight over whether women who need CS under the program actually receive the care that they need.

**Electronic supplementary material:**

The online version of this article (10.1186/s12939-019-0922-5) contains supplementary material, which is available to authorized users.

## Introduction

Cesarean section (CS) is a key emergency obstetric care signal (EmOC) function that has the potential to reduce maternal and neonatal mortality [1]. The World Health Organization (WHO) has recommended that CS rates should be between 10 and 15% [2] as CS rates above 16% have not been associated with lower maternal mortality (MM) [3]. However, other researchers have argued that the WHO recommendation on CS rates might be too low [4]. For example, Molina et al. found that CS rates below 19% but higher than 15% were also associated with lower MM [4].

CS rates vary significantly between geographical settings, ranging from less than one % in Chad to 45.9% in Brazil [5]. Despite this variation, CS rates are not static. Studies from southern Asia, Sub-Saharan Africa and Latin-America reported an increasing trend in the percentage of CS performed overtime [6, 7]. Voguel et al. using data from two multi-country WHO studies reported that the overall CS rate in 21 countries increased from 26.4% in 2004–2008 to 31.2% in 2010–2011.

### CS rates in Gujarat state India

Over the last 20 years, India has experienced a significant rise in institutional childbirth [8] which has also increased CS rates from two % in 1995 [6] to 19.3% in 2010 [7]. This phenomenon has been driven by increasing CS rates largely among the wealthiest part of the population, [6] who live mainly in urban settings. Gujarat state have also followed this trend with the percentage of CS increasing from three to eight % during the 1992–2006 period [9]. One of the factors explaining this phenomenon is the private sector dominance on the provision of obstetric care for all socioeconomic strata. In 2012, 70% of all births among poor/tribal Guajarati women occurred in private facilities which had five times the number of obstetricians per 1000 births than public facilities [10]. Overall, private facilities have significantly better obstetric care functionality (higher number of EmOC signal functions) than public ones [10].

### The Chiranjeevi Yojana public-private partnership program (CY PPP) and CS rates

Maternal access to comprehensive EmOC (including CS) in Gujarat has risen over time due in part to the Chiranjeevi Yojana public-private partnership program (CY PPP) led by the state to facilitate poor and tribal women’s access to EmOC [11, 12]. The CY PPP was a response to the extreme shortage of obstetricians in the free of charge public health care system which was unable to deliver EmOC effectively [13] forcing women to use private health facilities. Obstetric services in these facilities are accessed through out-of-pocket payments that are often prohibitive for poor and tribal women.

Under the CY PPP, the state pays private obstetricians a fixed sum (380,000 Indian rupees or $6000) per 100 births among eligible women living below poverty line (BPL) or belonging to scheduled tribes (ST). This sum was calculated based on an assumption that 85% of pregnancies would result in normal uncomplicated births and 15% would experience a complication, half of those requiring a CS [13].

The payment is fixed regardless of the actual number of CS performed per 100 births. This was intended to act as an embedded disincentive for unnecessary CSs [11, 13] by private obstetricians. This is an important feature as a number of studies from different parts of the world have reported higher CS rates in private for profit facilities [14, 15] arisen from supplier induced demand [16] stemming from differential payments for vaginal and cesarean births.

### Rationale

Increasing equitable access to comprehensive EmOC (including CS) is vital to decrease maternal morbidity and mortality especially among disadvantaged populations. The Guajarati CY PPP, with its embedded disincentive for excessive CS, has aimed to increase equity in the provision of EmOC among poor and tribal women without fostering unnecessary CS. However, it is unclear whether CS rates differ among eligible women who give birth within and outside the CY PPP program. In addition, no population-based studies have been conducted assessing whether the program’s incentive based on a predefined fixed CS rate assumption has influenced the performance of CS by the private obstetricians enrolled in the CY PPP.

In this paper, we aim to answer these questions by: 1. assessing the predictors of CS among eligible women giving birth within and outside the program and, 2. measuring the CS rates among beneficiaries of the CY PPP and comparing these rates with eligible women who give birth in non-CY private sector and public facilities.

The results of this paper are a first report studying the effectiveness of an embedded disincentive for cesarean section in a large public private partnership to promote institutional birth. Given the evidence of supplier induced demand in rising CS rates in the private sector, these results have important implications for similar public private partnerships being designed or implemented in other low-and middle-income settings.

## Methods

### Study design and study period

This community-based panel study is nested within a larger project [17] studying two large scale programs promoting institutional childbirth in India. In Gujarat, the project was implemented in three purposively selected districts (Dahod, Sabarkantha, and Surendranagar). The districts were selected because they cover diverse geographic and socioeconomic areas of the state (see Additional file 1: Table S1). Data collection was conducted between July 2013 and November 2014.

### Setting

Gujarat state (population of 60.4 million, 57.4% rural, 79% literate and 31.6% below poverty line, 21% tribal) [18, 19] is located in western India. The state is divided into 33 administrative districts, each with a population of between one-two million. Institutional childbirth is high in Gujarat relative to the rest of the country. In 2012, 80% of poor/tribal Guajarati women gave birth in a public or private institution [20]. The maternal mortality ratio in 2012 was 122 per 100,000 live births, which was lower than India’s average for the same year (178 per 100,000 live births) [21]. In the same year, the neonatal mortality rate was 28 per 1000 live births, similar to the Indian national average (29 per 1000 live births) [22].

### Sampling procedure

**Fig. 1:**
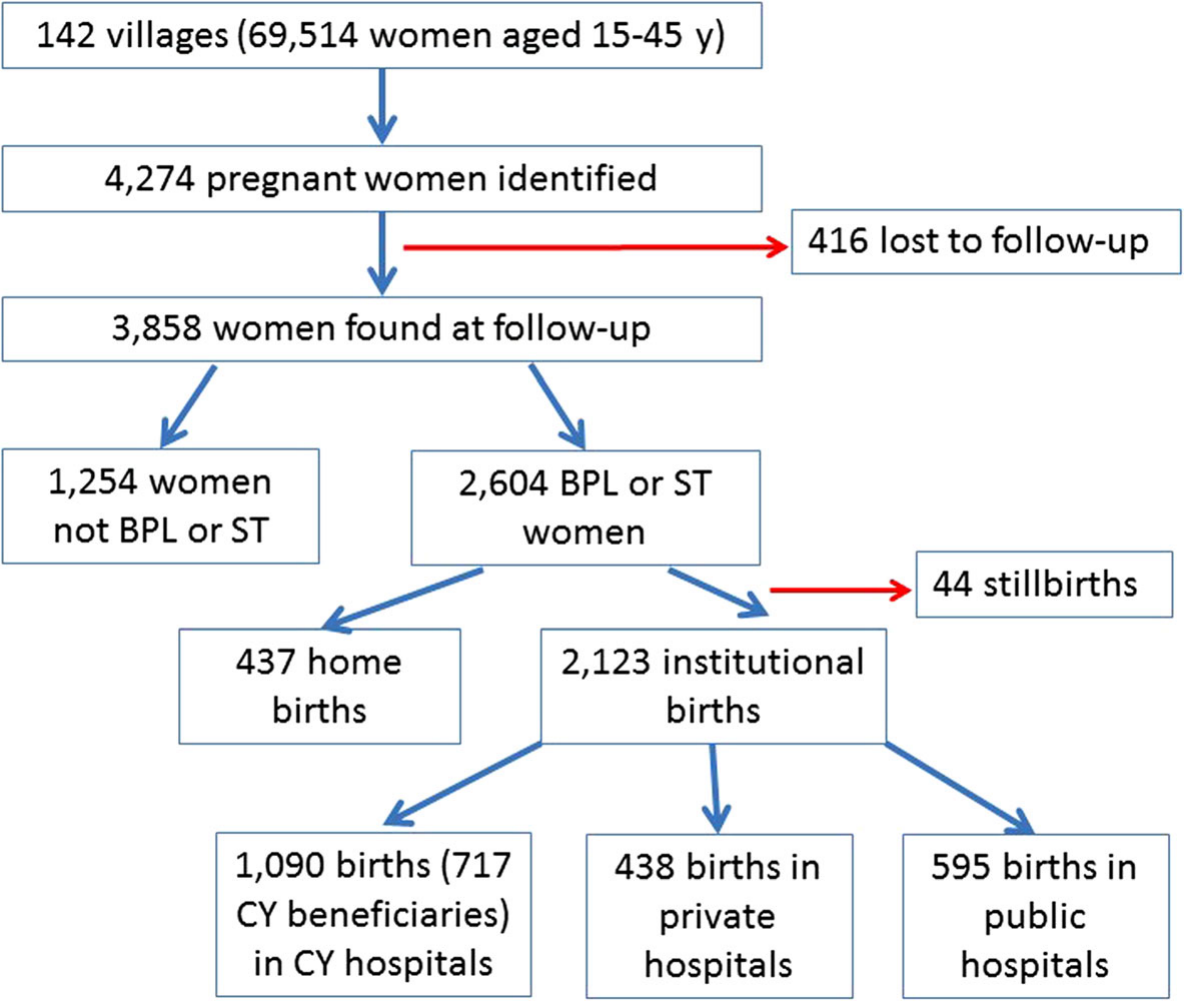
Sample flow chart

The sampling procedure is described in Fig. 1. First, villages with a population between 1000 and 2500 (of which at least 40% were BPL according to 2001 government census) in the three districts were selected (*n* = 142). Then all pregnant women who were expected to give birth during the study period (July 2013–November 2014) in these villages were identified through a household listing. Once identified, the pregnant women were interviewed by trained staff about socio-demographic and obstetric data. Subsequently, these same women were interviewed during the first 28 days after birth to gather information on the place and type of birth, intra-partum and postpartum morbidity.

Two thousand six hundred and four BPL or ST women completed both the pre-birth and post-birth questionnaire. Of those, we excluded 437 women who gave birth at home and 44 women who had stillbirths. The remaining 2123 women (81.5%) gave birth in a facility and were included in the subsequent analyses (Fig. 1).

### Measurements

The main outcome measure was whether the woman had a vaginal or a CS birth. CS rates were defined as the number of CS performed in a defined population during a reference period divided by the number of live births in the same reference period expressed as a percentage [23].

The main exposure assessed was CY PPP beneficiary status. A woman was classified as beneficiary if she fulfilled all of the following characteristics: a) gave birth in a private-CY accredited facility, b) had a BPL card or belonged to ST caste, and c) received a free or partially free child birth.

Other data collected comprised maternal socio-demographic and obstetric variables. The former included: age (years), education (years), religion (Hindu, Muslim, and Christian), district of residency (Dahod, Sabarkantha, or Surendranagar) and caste. Caste was divided into three groups: Scheduled tribes (ST), Scheduled caste (SC) and others. Scheduled tribes are a list of tribes in India’s constitution, who are considered disadvantaged social groups and receive positive affirmative action from the Indian government [24].

We also recorded women’s below poverty line status (BPL, identified by a state issued card). BPL status is assigned by the Indian government to families with earning below a certain threshold. It indicates economic disadvantage; holders of an official BPL card are granted access to government subsidies and benefits. Although most women included in our study had a BPL card, we further classified them based on household wealth. This was used as a proxy measure for women’s wealth status. A household wealth index based on household assets, the materials used for the construction of the family’s house, land ownership and sanitation provisions was constructed using principal component analysis and then divided in quintiles [25].

Obstetric data collected included the number of previous pregnancies, number of antenatal care visits for the index pregnancy, self-reported maternal complications for the index pregnancy (bleeding, high blood pressure, > 12 h labor, mal-presentation, anemia, or convulsions) and place of childbirth (public, private and CY PPP facilities). For analysis, public facilities were categorized as one group.

### Analysis

The data was analyzed using Stata v12 (StataCorp, College Station, Texas). Uni-variate, bivariate and multivariable statistics were used to describe and compare the data. Pearson chi-square and t-tests were used to compare bivariate differences between groups.

Multivariable logistic regression was used to obtain adjusted odds ratios (AOR) and 95% confidence intervals (CIs) for the association between our outcome variable (CS) and the main exposure (CY beneficiary status). Logistic regression was used due to the dichotomous nature of the outcome variable. Pseudo r square and Hosmer-Lemeshow goodness-of-fit test are also described to to assess the model’s fit.

Age, education, caste, number of previous pregnancies, number of antenatal care visits, household wealth, self-reported maternal complications during pregnancy, place of the childbirth and district of residency were included as co-variates since they have been significantly associated with CS in previous studies [6, 14, 15, 10]. BPL ownership was consider a proxy for household wealth and was excluded from multivariable models. Religion was also excluded since 99% of the sample reported being Hindu.

### Sample power analysis

We did a post-hoc assessment of the study power to estimate differences between in CS incidence rates among CY beneficiaries and non-beneficiaries within private CY accredited facilities (*n* = 1090). Given a two-sided 95% CI, a sample of exposed (CY-beneficiaries) of 717, a sample of non-exposed (non-beneficiaries) of 373 and CS risks of four point seven % and 12.3% respectively the study has a 99.5% power to assess statistical differences between these two groups.

## Results

### Maternal characteristics

Maternal characteristics are described in Table 1. Mean maternal age was 24.1 years (SD = 3.54). Half gave birth in private-CY accredited facilities (1090/2123) and eight % reported having a complication during pregnancy (171/2123). Nine in ten women had a BPL card. Thirty four percent (717/2123) of the sample were CY program beneficiaries. Of the non-beneficiaries (*n* = 1406), 42% gave birth in public facilities (595/1406), 31% in private non-CY accredited facilities (438/1406) and 27% in private-CY accredited facilities (373/1406) (Fig. 1). Figure 1 indicates that 66% (717/1090) of eligible women who went to a CY program accredited facility did become program beneficiaries, while a third did not.

**Table 1:** Maternal characteristics, facility type and district stratified by C-section occurrence

Characteristics	All births	C-section No	C-section Yes
	*n* = 2123	*n* = 1959	*n* = 164
	n (%)	n (%)	n (%)
Age. Mean (SD)^a^	24.1 (3.54)	24.2 (3.58)	23.4 (2.97)
Education. Mean (SD)	5.1 (4.54)	5.1 (4.53)	5.5 (4.57)
Caste ^a^			
ST	860 (40.5)	829 (42.3)	31 (18.9)
SC	157 (7.4)	138 (7.0)	19 (11.6)
Others	1106 (52.1)	992 (50.7)	114 (69.5)
Number of pregnancies Mean (SD)^a^	1.15 (1.30)	1.19 (1.32)	0.71 (0.91)
Antenatal care visits			
3 or more ^a^	1558 (73.4)	1418 (72.4)	140 (85.4)
BPL card. Yes ^a^	1920 (90.4)	1762 (89.9)	158 (96.3)
Wealth quintiles^a^			
Lowest	360 (17.0)	347 (17.7)	13 (7.9)
Low	383 (18.0)	364 (18.6)	19 (11.6)
Middle	433 (20.5)	400 (20.4)	33 (20.1)
High	439 (20.6)	406 (20.7)	33 (20.1)
Highest	508 (23.9)	442 (22.6)	66 (40.3)
Complications. Yes^a^	171 (8.0)	103 (5.3)	68 (41.4)
District ^a^			
Dahod	775 (36.5)	739 (37.7)	36 (21.9)
Sabarkantha	738 (34.8)	681 (34.8)	57 (34.8)
Surendranagar	610 (28.7)	539 (27.5)	71 (43.3)
Facility type^a^			
Public	595 (28.0)	571 (29.1)	24 (14.6)
Private-CY	1090 (51.4)	1010 (51.6)	80 (48.8)
Private	438 (20.6)	378 (19.3)	60 (36.6)
CY beneficiary. Yes^a^	717 (33.8)	683 (34.7)	34 (20.7)

^a^Chi2 test or t-test *p* < 0.05

### CS rates among eligible women within and outside the CY program

**Fig. 2:**
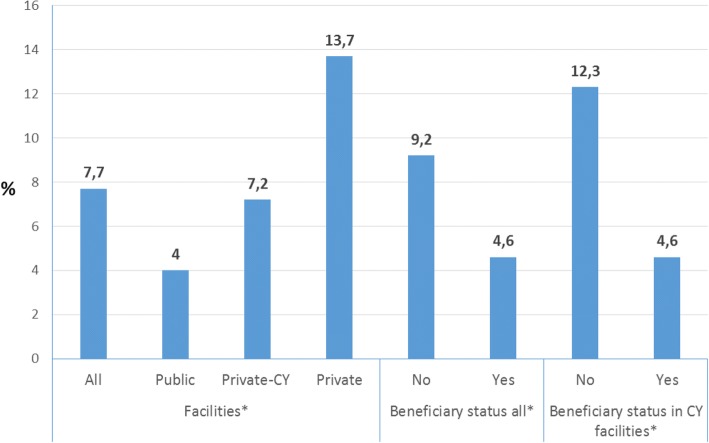
Percentage of women who had a cesarean-section in different sub-samples; *p < 0.05

Overall seven point seven % (164/2123) of the all women in the study had a CS. The CS rate in non-beneficiary women was almost twice as high (at nine point two % compared to women who gave birth under the CY program (four point six % (*p* < 0.05) (Fig. 2). CS rates varied widely by the place of delivery. Women who gave birth in public facilities had significantly lower CS rate (four %) than those giving birth in accredited CY-private (seven point two % or non-accredited private facilities (13.7%) (*p* < 0.05, Fig. 2). The overall proportion of CS births within CY accredited facilities was seven point two %, however when this was disaggregated by beneficiary status, the rates among non-beneficiaries was similar to that among women giving birth in the private sector at 12.2% (four point six % among beneficiaries) (*p* < 0.05, Fig. 2).

### Predictors of CS rates among vulnerable women in Gujarat

Bivariate analysis showed that women who had a CS were younger, less likely to be tribal caste, lived in Surendranagar district, were richer, gave birth in private facilities, had a complication, had more antenatal care visits, had a BPL card and were more likely to be classified as non-CY beneficiaries than those who did not have a CS (Table 1, *p* < 0.05).

### Multivariable analysis

First we describe the results of our multivariable analysis with the 2123 women in our cohort. The odds of having a CS were higher among non-tribal women, primiparas, those giving birth in private-accredited CY or private-non accredited facilities and those reporting any complication during pregnancy (Table 2, *p* < 0.05). CY beneficiaries had 62% (AOR 0.38, 95% CI 0.22–0.44) lower odds of having a CS than to non-beneficiaries (Table 2).

**Table 2:** Risk factors for Cesarean-section in different sub-groups, adjusted Odds Ratios (AOR) and 95% confidence intervals (CI) shown

Characteristics	All *n* = 2123	CY-beneficiaries No *n* = 1406	CY-beneficiaries Yes *n* = 717	Only women delivering at CY facilities. *n* = 1090
	AOR 95% CI	AOR 95% CI	AOR 95% CI	AOR 95% CI
CY beneficiary. Yes	0.38 (0.22–0.44)	–	–	0.40 (0.24–0.67)
Age (years)	1.05 (0.98–1.13)	1.01 (0.93–1.10)	1.18 (1.03–1.34)	1.11 (0.99–1.23)
Education (years)	1.00 (0.96–1.04)	1.01 (0.96–1.06)	0.96 (0.86–1.06)	0.97 (0.91–1.03)
Caste				
ST	1.00	1.00	1.00	1.00
SC	2.11 (1.01–4.43)	1.44 (0.61–3.40)	5.88 (1.29–26.71)	4.05 (1.44–11.04)
Others	1.89 (1.10–3.25)	1.46 (0.78–2.74)	3.48 (1.10–10.99)	2.54 (1.20–5.35)
Number of pregnancies (unit)	0.64 (0.50–0.82)	0.70 (0.53–0.91)	0.49 (0.28–0.84)	0.50 (0.35–0.73)
Antenatal care visits				
0–2	1.00	1.00	1.00	1.00
3 or more	1.67 (1.02–2.75)	1.68 (0.97–2.92)	1.97 (0.60–6.46)	1.75 (0.82–3.59)
Wealth quintiles				
Lowest	1.00	1.00	1.00	1.00
Low	1.01 (0.46–2.19)	1.20 (0.48–2.99)	0.60 (0.12–2.84)	0.80 (0.30–2.16)
Middle	1.28 (0.63–2.61)	1.58 (0.69–3.62)	0.58 (0.12–2.67)	1.16 (0.48–2.81)
High	1.15 (0.54–2.43)	1.16 (0.48–2.78)	1.15 (0.24–5.42)	0.81 (0.29–2.22)
Highest	1.35 (0.63–2.87)	1.51 (0.63–3.59)	0.83 (0.15–4.57)	0.61 (0.21–1.75)
Complications. Yes	10.71 (7.22–15.9)	10.18 (6.50–15.92)	13.18 (5.54–31.38)	6.00 (3.30–10.91)
District				
Dahod	1.00	1.00	1.00	1.00
Sabarkantha	1.05 (0.60–1.82)	1.10 (0.58–2.09)	1.29 (0.37–4.54)	0.83 (0.38–1.80)
Surendranagar	1.13 (0.59–2.18)	1.19 (0.57–2.46)	1.30 (0.25–6.70)	1.67 (0.64–4.38)
Facility type				
Public	1.00	1.00	–	–
Private-CY	3.16 (1.80–5.53)	2.95 (1.69–5.17)		
Private	2.55 (1.46–4.44)	2.56 (1.46–4.49)		
Pseudo r2	0.21	0.15	0.24	0.19
Hosmer-Lemeshow GOF test	*P* = 0.97	*P* = 0.96	*P* = 1.00	*P* = 0.99

When we studied predictors of CS separately among CY beneficiaries and CY non beneficiaries (Table 2), these were similar in both groups (parity and complication reported). However among CY beneficiaries, tribal women and older women had lower odds to have a CS birth than women belonging to other subpopulations (Table 2, *p* < 0.05).

In a separate model of predictors of CS among women giving birth only in accredited -CY facilities, we found that CY program beneficiaries had lower odds of having a CS birth than non-beneficiary women (paying clients) (AOR 0.40, 95% CI 0.24–0.67). Other significant predictors were similar to those in the models described above (see Table 2, *p* < 0.05).

## Discussion

Our study focuses on vulnerable women (ST/BPL) giving birth within or outside the large CY PPP to promote institutional births and improve access to EmOC among this group. Our main findings show that CS rates for CY beneficiaries (four point six %) were lower than the provision made for these in the financial reimbursement package (seven %) paid by the state to private obstetricians under the PPP. In addition, after adjusting for possible confounders CY beneficiaries had lower odds of having a CS among non-beneficiaries. This difference persisted even within the subgroup giving birth within and outside the program only in accredited CY facilities.

### An interpretation of our finding of lower CS rates among CY beneficiaries

This key finding suggests that CY beneficiaries have lower access to this comprehensive EmOC signal function than non-beneficiaries. Our previous studies in this setting provide some support for this hypothesis. Ganguly et al. [26] found that private obstetricians participating in the CY PPP considered the CY PPP remuneration for CS insufficient resulting in a loss of income. In addition, they perceived that the 7 % CS rate stipulated in the CY PPP was unrealistic suggesting that significantly higher rate (15–20%) needs to be considered. Bogg et al. also reported that the CS rate in the CY PPP declined from six point seven % in 2007 to four point three % in 2011 [27].

Given the marketization of healthcare in this context, it is possible that private obstetricians will want to maximize pecuniary gains, and so keep CS births under the program as low as possible (or at least as close to the assumption in the reimbursement package) to make CY participation financially advantageous. However, since our study did not gather clinical data, it is not possible to determine whether all women in the program who had a clinical indication for a CS obtained one; or if a clinical indication for CS resulted in an eligible women being moved out of the program (sometimes even in the same facility). While the higher rate of CS among eligible non beneficiaries even in the same facilities suggests this might be the case, further studies are needed to investigate this.

Another unexpected finding of our study supporting the aforementioned hypothesis is that CS rates among women delivering in public facilities (four %) where almost identical to CS rates among CY beneficiaries (four point six%). This is striking since the latter give birth in facilities with five times as many obstetricians and significantly higher obstetric care functionality [10] than public facilities. If public and CY PPP CS rates are similar, then the PPP is not improving vulnerable women’s access to comprehensive obstetric care, which would make the benefit of implementing the CY PPP debatable other than providing some increase in geographic coverage.

### Maternal risk factors for CS

Regardless of place of delivery and other possible confounding factors, women who reported complications during pregnancy (bleeding, high blood pressure, > 12 h labor, mal-presentation, anemia, or convulsions) had higher CS rates than those who did not. This, as expected, is in line with the results of studies conducted in South Asia [15] and Australia [14]. Our results also highlighted that the number of previous pregnancies decreased CS risk and this is in line with the findings of one study conducted in Jamaica [28].

Maternal caste (belonging to SC or other castes) was significantly associated with higher CS rates than ST women. This finding is not completely unexpected since castes in India are part of a complex system of social stratification where some groups (such as ST caste) are more socially isolated and impoverished whereas others (such as SC or other castes) have relatively more access to societal resources and networks [29].

### Strengths and limitations

Our study has important limitations that need to be acknowledged. Our study did not gather information on maternal exposure to previous CS which has been shown to increase CS rates.

A limitation of our study and other population-based studies is that gathering reliable clinical details of events leading up to childbirth from the women is difficult. Thus, we were unable to adjust for these potential confounders (gestational age at birth, spontaneous or induced labor, elective or emergency CS, etc.). Also, our complications variable is self-reported. There is evidence that indicated that self-reported complications are often overestimated [30].

Without access to clinical records, we were also unable to establish whether beneficiaries who needed CS received this or whether they received CS but were denied access to the program. This limitation restricts our ability to objectively comment upon why CS rates are so low in the program, though it does provide an initial quantitative suggestion that needs more in-depth exploration. In spite of all the aforementioned limitations, our study also has important strengths. Our paper firmly establishes that CS rates in the PPP are lower than private obstetricians receive reimbursement for. Also that CY beneficiaries have fewer CS births than non-CY beneficiaries.

## Conclusions, policy and practice implications

The Gujarat government is trying to ensure access to EmOC (including CS) for its vulnerable population through this PPP. The idea of an embedded disincentive is a novel one, and appears to work, though one could wonder if it works ‘over-efficiently’ by depriving some women who need CS from receiving one under the program.

The solution to this problem does not lie in removing the embedded disincentive as that opens up the possibility of very high CS rates because of supplier induced demand as seen in a neighboring state [27]. The state needs to possibly revisit and review what is happening in the program periodically, and have oversight over whether women who need CS under the program actually receive these or are moved out of the program because of the way the reimbursement under the program is structured. In addition, a focus on wider indicators to determine provider performance (still births, neonatal deaths, postnatal complications) rather than a narrow focus on the provision of 48 h intra-partum service is necessary.

## Additional file

Additional file 1: **Table S1.** Characteristics of the study districts. (DOCX 14 kb)
