# Supplementary material for: Caesarean sections in the in the context of the Chiranjeevi Yojana public private partnership program to promote institutional birth in Gujarat, India; does the embedded disincentive for caesarean section work?
Source: Int J Equity Health. 2019 Jan 24;18:17. doi: 10.1186/s12939-019-0922-5 (PMC6345034; doi:10.1186/s12939-019-0922-5)
Supplement: Supplementary file 1 — Table S1. Characteristics of the study districts. (DOCX 14 kb) [file 12939_2019_922_MOESM1_ESM.docx]

Additional file 1: Table S1. Characteristics of the study districts

| Characteristics | Dahod | Sabarkantha | Surendranagar | Gujarat |
| --- | --- | --- | --- | --- |
| Population (2011)^*^ | 2,127,086 | 2,428,589 | 1,756,268 | 60,383,628 |
| Crude birth rate x 1000 population (2011)‡ | 30.2 | 28 | 23 | 22.7 |
| Proportion rural population (2011)* | 91% | 85% | 72% | 57.4% |
| Proportion literate population* | 58.8% | 75.8% | 72.1% | 79.3% |
| Proportion BPL population^†^ | 71.6% | 32.9% | 46.5% | 40.3% |

^*^Sample Registrar of India (2011). Districts of Gujarat. from http://www.census2011.co.in/census/state/districtlist/gujarat.html. Socio Economic Survey 2002-03. Add-on lists 2008-09 [database available online].  ^†^Commissionerate of Rural Development, Gujarat. http://ses2002.guj.nic.in/. Accessed 15 January 2015. ‡Vital statistics Division Government of Gujarta. Civil Registration System in Gujarat, Annual Statistical Report 2010. Gandhinagar, 2011
